# Supermicrosurgical treatment for lymphedema: a systematic review and network meta-analysis protocol

**DOI:** 10.1186/s13643-022-01885-9

**Published:** 2022-02-01

**Authors:** Patrick A. Will, Zhenzhen Wan, Svenja E. Seide, Juan Enrique Berner, Ulrich Kneser, Emre Gazyakan, Christoph Hirche

**Affiliations:** 1 Department of Hand, Plastic, and Reconstructive Surgery, Microsurgery, Burn Centre, BG Trauma Center Ludwigshafen, Ludwig Guttmann Str. 13, 67071 Ludwigshafen am Rhein, Germany; 2 Medical Faculty of the University Heidelberg, Heidelberg, Germany; 3 Institute of Medical Biometry and Informatics, University of Heidelberg, Heidelberg, Germany; 4 Kellogg College, University of Oxford, Oxford, UK; 5 Department of Plastic Surgery, The Royal London Hospital, London, UK; 6 Department of Plastic, Hand, and Reconstructive Microsurgery, BG-Trauma Hospital Frankfurt am Main, Frankfurt am Main, Germany

## Abstract

**Background:**

Lymphedema is a condition that affects up to 130 million subjects worldwide. Since it is related to several complications and a significant reduction in terms of quality of life, it is a heavy burden not only to the patients but also for the healthcare system worldwide. Despite the development of supermicrosurgery, such as vascularized lymph node transfer (VLNT) and lymphovenous anastomosis LVA, the indications and outcomes of these complex groups of interventions remain a controversial topic in the field of reconstructive plastic surgery.

**Methods:**

This systematic review and network meta-analysis aims to assess the evidence of outcomes of LVA and VLNT in patients with lymphedema. Secondary aims of the project are to determine if for any outcomes, LVA or VLNT is superior to conservative therapy alone, and whether the available evidence favors any kind of supermicrosurgical interventions for lymphedema patients. This study will include original studies of patients with lymphedema on the extremities indexed in PubMed, EMBASE, CENTRAL, PASCAL, FRANCIS, ISTEX, LILACS, CNKI, and IndMED that reported microsurgery (supermicrosurgery) of all techniques aiming the re-functionalization of the lymphatic system. As comparators, mere observation, conservative treatment of any kind, and the other subgroups of supermicrosurgery are planned. The primary outcome of this systematic review and network meta-analysis is the difference of the limb volume, while the secondary outcomes of interest will be erysipelas rates, major and minor complications, postoperative necessity of continuous compression garments, and patient satisfaction, measured by already published and validated scores for quality of life.

**Discussion:**

We will provide an overview and evidence grade analysis of the scientific literature available on the effectiveness of the subcategories of supermicrosurgical interventions for lymphedema.

**Supplementary Information:**

The online version contains supplementary material available at 10.1186/s13643-022-01885-9.

## Background

Lymphedema is characterized by the development of localized swelling as a result of a compromised lymphatic system [1]. Primary lymphedema occurs when there is congenital dysfunction of the lymphatic system (LS) caused by isolated mutations or chromosomal aberrations [2]. Secondary lymphedema (SL) is the result of an injury to the LS subsequent to bacterial or parasitic infections, malignancy, radiation, trauma, inflammation, and medications [3].

Despite major improvements in public health and access to healthcare, parasitic infections remain the main global cause of SL, such as filariasis or onchocerciasis [4]. The overall prevalence of infectious SL has been appraised to exceed the 200 million cases worldwide [4]. In the developed world, the leading cause of lymphedema is iatrogenic, following oncological treatment. It is estimated that 10–70% of the patients surviving a solid malignancy will develop lymphedema, depending on the extent of lymphatic surgery required and adjuvant therapies [5].

The currently accepted system of staging lymphedema is the one proposed by the International Society of Lymphology (ISL) [6]. At stage 0, there are no features of clinical lymphedema, yet damage of the lymphatic system can be detected by imaging. In stage 1, an early accumulation of fluid is seen, but subsides with limb elevation or compression [6]. If limb elevation does not reduce tissue swelling and pitting edema is evident, then lymphedema is classified as stage 2 [6]. Stage 3 encompasses lymphostatic elephantiasis, in which the skin does not pit because of trophic skin changes such as fibrosis, acanthosis, fat deposits, and warty overgrowths; that also may lead to the loss of contour and anatomic shape of the affected extremity [6].

In early stages, compression therapy is still the workhorse approach for lymphedema. The goal of compression garments is to increase interstitial pressure and decrease capillary filtration [7]. The literature has shown that patients with lymphedema treated with compression garments in early stages are able to lose a mean excess of 1700cc or 20% of the limb volume within 2 weeks [8]. Low-stretch bandage, manual lymph drainage, regular exercise, and skincare are usually associated with compression, as part of what is called complex decongestive therapy [7, 9]. Since the 1980s, this has been the gold standard for lymphedema. However, this modality only addresses the signs and symptoms derived from excessive edema formation, without treating the underlying lymphatic dysfunction or pathophysiology of disease progression.

Functional lymphedema surgery involves two sets of techniques: lymphovenous anastomosis (LVA) and vascularized lymph node transfers (VLNT) [10]. The anastomosis of lymphatic vessels to small cutaneous veins or afferent vascular package of lymph nodes is considered supermicrosurgery, as it involves suturing structures with a diameter of less than 0.8mm [11]. LVA surgery aims to create a bypass by which fluid excess can be drained into the venous system at different levels in the affected extremity. Reports of long-term average volume reduction range from 2.4 to 69% for LVAs [12, 13]. The introduction of intraoperative indocyanine green (ICG) navigation to identify the LV in situ and test anastomosis patency has refined this procedure further and added another layer of technical complexity to an already challenging procedure [14]. VLNT comprises the transplant of vascularized autologous lymph nodes from a donor site and anastomosis to recipient vessels in the affected limb. It has been theorized that the transferred lymph nodes act as a sponge that absorbs the excess of lymph fluid while generating a local lymphatic system de novo by lymphangiogenesis [15–17]. Despite that these proposals have not been proven, studies have shown an average volume reduction from 7.13 to 74.5% and a reduction for the necessity for long-term compression therapy [18, 19], although it has been argued that there is a lack of reproducibility of studies obtaining these outcomes [20].

Due to the absence of strong evidence concerning the effectiveness of LVAs and VLNTs, clear guidelines concerning the use of these procedures are missing. Only two older clinical practice guidelines discussed indications for surgery in lymphedema. While the International Lymphedema Framework guideline in 2012 concluded that “peer-reviewed published literature on the surgical treatment of Lymphedema indicate that these procedures are promising for select groups of patients” [21], the CREST guideline of 2008 declared that “Surgery is not currently recommended for the management of this condition in Northern Ireland” [22]. Recently, microsurgical and plastic surgical societies have proposed consensus recommendations for the supermicrosurgical treatment of lymphedema to orientate clinicians looking after patients with lymphedema [23]. Here, it was also formulated that further evidence-based recommendations are of imperative necessity.

## Methods/design

The following protocol for this systematic review and network meta-analysis adheres to the Preferred Reporting Items for Systematic review and Meta-Analysis Protocols (PRISMA-P) guidelines [24], in accordance with the PRISMA-NMA statement [25] and with a methodology recommended by the Cochrane Handbook [26]. The completed PRISMA-P checklist is provided in the supplementary material (Supplementary file 1). This study could not be registered in the Prospective Register of On-going Systematic Reviews (PROSPERO) because PROSPERO does not currently accept registrations for scoping reviews, literature reviews, or mapping reviews (Supplementary file 1). Any variances from the methods described in this protocol will be reported and discussed in the final systematic review and network meta-analysis.

### Aim

This systematic review and network meta-analysis is intended to assess the evidence of outcomes of two types of supermicrosurgical interventions (LVA and VLNT) in patients with lymphedema. Secondary aims of the project are to determine if for any outcomes, LVA or VLNT is superior to conservative therapy alone and whether the available evidence favors any kind of supermicrosurgical interventions for lymphedema patients. An additional aim is to determine the areas where further evidence-based oriented reports are missing.

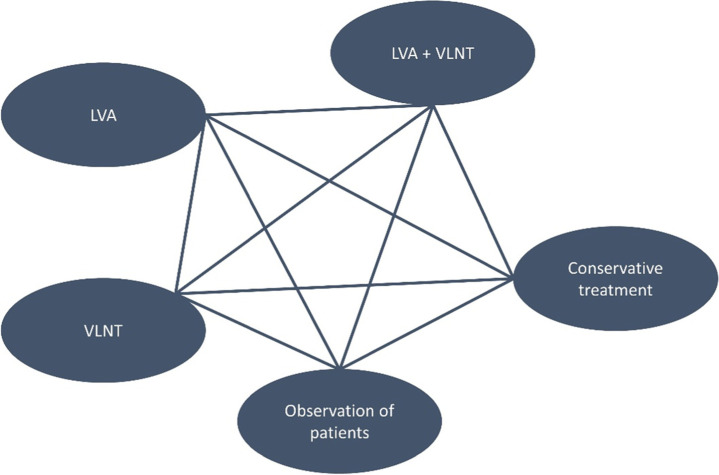



### Eligibility criteria

The eligibility criteria have been established following the Population, Intervention, Comparator, Outcomes, and Study Design (PICOS) format recommended by the Cochrane Group [27]. These are described in detail below and summarized in Table 1.

**Table 1:** Summary of the inclusion and exclusion criteria of the planned systematic review and network meta-analysis

	Inclusion criteria	Exclusion criteria
**1. Population**	Patients with lymphedema on the extremities without exclusion of demographic factors	Intraperitoneal lymphatic diseases or only genital or head and neck lymphedema
**2. Intervention and comparators**	Microsurgery (supermicrosurgery) of all kinds and techniques aiming the re-functionalization of the lymphatic system	Microsurgical treatments of complications of lymphedema or Microsurgical interventions of tissue-engineered constructs and implants
		Cohorts or studies with ablative operative treatments
**3. Study design**	Original studies with no restrictions in language, years, or geography.	Preclinic studies, case reports, consensus papers, editorials, reviews, and meta-analysis
		Original publications of the same researcher’s group with overlapping time frame and no evidence that the cohorts described are different
		Original reports on preprinted servers

### Population

This study will include patients with lymphedema on the extremities without exclusion of demographic factors (age, gender, ethnicity, or socio-cultural settings). No specific subpopulations of secondary lymphedema will be excluded, and if possible, these subpopulations will be pooled according to the stage of the disease (following the classification of the ISL [6]) and/or the etiology of the lymphedema). Patients with intraperitoneal lymphatic diseases or only genital or head and neck lymphedema will not be included. In order to minimize selection bias, all patient cohorts where the main intervention was the microsurgical treatments of complications of lymphedema, such as lymphatic cysts, lymphorrea, lymphocele, or lymphangiosarcoma, will be excluded. It is possible to assume transitivity of the population studied because, despite expected ethnical differences, most patients will have a similar distribution of age and lymphedema etiology. Socio-cultural differences among the population or ethnical variations are of minor importance for the transitivity assumptions since the access to healthcare, lifestyle, and co-morbidities will be distributed similarly. This is because the studied and intervened population will be of developed countries with a healthcare system able to provide complex supermicrosurgical procedures.

### Intervention

The intervention of interest is microsurgery (supermicrosurgery) of all kinds and techniques aiming the re-functionalization of the lymphatic system. Supermicrosurgery will not be strictly defined during this network meta-analysis based on the traditional definition as procedures with anastomosis of vessels smaller than 0.8mm [11], but as a concept of microsurgical treatment for lymphedema. This broader definition will be considered during the search strategy to avoid unintentional exclusion of suitable publications. As described above, the microsurgical treatment of lymphedema can and will be divided during this study roughly in two groups: vascularized transfer of autologous lymph nodes from a donor side (intra- or extra-abdominal VLNT and soft tissue flaps including lymph nodes) or lymphatic shunts or bypasses (lymphovenous anastomosis or lympholymphatic bypass). Combined interventions will be included and, if possible, pooled into an extra intervention group. Microsurgical interventions of tissue-engineered constructs and implants as shunts/bypass of the lymphatic system will be excluded. Dose-dependent interventions might be relevant (like the amount of lymph nodes transferred or the number of LVA performed per intervened subject). Any dose-dependent information will be considered and extracted. After matching dose-dependence of the interventions in the comparable subgroup of individuals, further comparisons might follow.

### Comparators

In randomized controlled trials, inactive and active control interventions will be included. These are respectively (1) mere observation—do nothing and (2) conservative treatment of any kind (compression garments, manual decongestive therapy, pneumatic compression, lymphatic drainage, etc.). Ablative operative treatments as liposuctions or excisional-and-grafting surgeries will not be considered as an active control group during this systematic review and network meta-analysis. No exclusion based on frequencies, duration, timing of delivery, or number of interventions will be considered. A pooled comparation of two groups, LVA and VLNT, will be performed during this systematic review and network meta-analysis. Trials including only inactive or active control trials or case series, without a microsurgical intervention group for lymphedema, will be excluded. Studies including merely a supermicrosurgical intervention group, if considered as appropriate after the risk of bias assessment (see the “Risk of bias assessment” section), will be pooled for further comparisons. There will be no exclusions based on the postinterventional follow-up period. To permit an analysis, the results of the follow-up period will be pooled arbitrary in four groups: (1) direct postoperative period—day 1 to 14, (2) early postoperative period (2nd week to 6th month), (3) late postoperative period (6th month to 2nd year), and (4) long-term postoperative period (after 2 years). If a trial reports multiple timepoints that fall into the same of our pre-defined categories, we will use the latest available timepoint in that category.

### Outcomes

The primary outcome of this systematic review and network meta-analysis is the difference of the limb volume, measured by water plethysmography, circumference variation, or mathematic volumetric calculations on different scales. The limb volume difference of the limbs with lymphedema at a pre- and postoperative timepoint or the comparison of the affected and intervened limb with the contralateral extremity will be included and pooled into subgroups. Secondary outcomes of interest will be erysipelas rates, major and minor complications, postoperative necessity of continuous compression garments, and patient satisfaction, measured by already published and validated scores for quality of life (Table 2). Subjective reports of satisfaction or the application of non-validated quality-of-life scores will not be considered. If possible, the primary and secondary outcomes will be polled and analyzed for the different stages of lymphedema, its etiology, and for each specific interventional subgroup.

**Table 2:** Overview of the primary and secondary outcomes of the planned systematic review and network meta-analysis

**Primary outcome**	Limb volume difference either (1) Pre- and postoperatively or (2) Comparison of the intervened limb with the contralateral extremity
**Secondary outcomes**	1. Erysipelas rates 2. Postoperative major and minor complications 3. Need of continuous compression garments after the intervention 4. Patient satisfaction, measured by validated scores for quality of life

### Type of studies to be included

Any original articles, which assess the outcomes of supermicrosurgery for lymphedema, either on its own or compared to other supermicrosurgical or conservative therapies, will be included. There will be no restrictions in language, years, or geography. Our exclusion criteria include preclinic studies, case reports, consensus papers, editorials, reviews, and meta-analysis articles. Also, original publications of the same researcher’s group with overlapping time frame might be excluded if the patients are not singularized as different cohorts. In such a scenario, the authors will be contacted to confirm that the patient cohorts of the articles in question are different. If this information is not available to us, the study with the highest evidence level or the largest series, in case of equal study design, will be included. The other article, or articles, will be excluded. Since preprint servers like MedRxiv are not peer reviewed, the corresponding grade of evidence cannot be certainly determined. Therefore, preprinted reports will not be considered.

### Search strategy

A comprehensive and reproducible electronic search will be conducted by four independent reviewers in multiple electronic databases including MEDLINE via PubMed, EMBASE via Ovid, Cochrane Central Register of Controlled Trials (CENTRAL), PASCAL, FRANCIS, ISTEX, Latin American and Caribbean Health Sciences Literature (LILACS), China National Knowledge Infrastructure (CNKI), and Indian Biomedical Research Database (IndMED). An example of the search strategy and key words to be used during the systematic review and network meta-analysis is provided in Table 3.

**Table 3:** Exemplary search strategies and key words that will be used for this systematic review and network meta-analysis

Search string for **MEDLINE via PubMed**	“Lymphedema” OR “Lymphedema” OR “Lyphatic edema” OR “Lymphatic oedema” OR “Elephantiasis” OR “Swelling” AND (“Microsurg*” OR “Supermicrosurg*” OR “Lymphoven*” OR “Lympholympha*” OR “Microlympha*” OR “Lymphoven*” OR “Shunt” OR “Bypas*” OR “Anastomo*” OR “Lymph node” OR “Lymph* flap” OR “Transfer” OR “Transplant*”)
Advance search for **CENTRAL via Cochrane**	#1 : MeSH descriptor: (Lymphedema) explode all trees #2 : (“Lymphoedea” OR Lymphatic edema” OR “Lymphatic oedema” OR “Elephatiasis” OR “Swelling):ti,ab,kw #3 : #1 OR #2 #4 : MeSh descriptor: (Microsurgery) explode all trees #5: (“lymphovascular” or “anastomosis” or “lymphovenous” or “lympholymphatic” or “supermicrosurgery” or “lymph node” or “transplant” or “transfer” or “shunt” or “bypass” or lymphatic flap”:ti,ab,kw #6: #4 OR #5 #7: #3 AND #6

Additionally, unpublished clinical trials will be searched by the same four reviewers independently at ClinicalTrials.gov, the International Standard Randomized Controlled Trial Number Register (ISRCTN), and the WHO International Clinical Trials Registry Platform (ICTRP). Of these trials, a back- and forward citation research will be performed by hand. Despite reviews, meta-analysis and systematic reviews will be excluded from further analysis; a manual screen of the references cited will be conducted to detect potentially overlooked articles. In order to identify all relevant studies, all the above-mentioned databases will be systematically searched from their inception with no language limitations.

### Data extraction

Two of the same review authors that performed the search will evaluate the studies, obtained and saved as abstracts in EndNote X9 (version X9.3.2, Clarivate Analytics, Philadelphia USA), and exclude independently the publications based on the inclusion and exclusion criteria. Then, the list of the remaining studies to be included will be compared and any difference of excluded articles will be solved by consensus with a third reviewer. The same reviewers will conduct a full-text screening and exclude again unsuited articles. Once again, the result of this process will be compared, and the differences solved by mutual agreement with a third team member.

A standardized data extraction spreadsheet will be developed a priori (Microsoft Excel, version 16.45, Microsoft, Washington, USA). The final studies to be included will be analyzed independently by three reviewers and the data extracted and filled into the Excel form. Inconsistencies will be resolved by unanimity after consultation with a fourth reviewing member. The following data will be extracted: year of publication, study location, type of study, cohort size, age, gender, follow-up period, risk factors for complications, supermicrosurgical intervention, control intervention, and the outcomes as outlined in the PICOS strategy (Table 1). Specific details of postoperative medications, physical rehabilitation, and lymphatic compression protocols will also be extracted, but a priori not analyzed. Sources of funding and declared conflict of interests will be collected as part of the risk of bias assessment. The authors will be contacted if there is data missing and the eligibility criteria or data description remains unclear following the article reviews. If data is consistently missing and remains unprovided by the respective authors, they will be imputated by using the informative missingness odds ratio (IMOR) method [28]. After the extraction occurred, some studies might still be excluded based on the bias risk (see the “Risk of bias assessment” section below). An overview of the studies excluded and included will be presented in a PRISMA flow diagram.

### Risk of bias assessment

After the extraction occurred, the included studies will undergo a risk of bias assessment and reporting quality by three review authors independently. If the bias of studies is evaluated as too high by any reviewer, the assessment will be discussed with the other reviewing authors and excluded or included by consensus. The results of the risk of bias assessment will be summarized narratively and graphically in the main body or the appendix of the resulting systematic review and network meta-analysis. Additionally, the risk of bias between studies will be assessed and presented as funnel plots. If a small-study effect is observed, we expect it to slightly overrate the positive outcomes of supermicrosurgery in the intervened population in contrast to the expected effect when broadly applied. As with any other complex surgical procedure, supermicrosurgery, it better performed in the highly specialized centers, that in turn publish the first cohort series or RCTs.

In detail, the risk of bias will be assessed using the Cochrane Handbook’s Risk of Bias revised tool (ROB2) [29] assessment tool for randomized controlled trials and graded as “low risk,” “high risk,” and “unclear” in each of the following areas: random sequence generation, allocation concealment, blinding of participants and personnel, blinding of outcome assessment, incomplete outcome data, selective reporting, and other bias. For non-randomized studies, the risk of bias will be evaluated by ROBINS-I (Risk of Bias in Non-randomized Studies-of Interventions) [30]. For case series, the bias inquiry will be addressed using the Critical Appraisal Checklist for Case Series provided by the Joanna Briggs Institute (JBI) [31].

### Data synthesis

We will summarize and describe each study narratively in tables and text by general characteristics of the study population, treatment, and design. All values that are relevant for quantitative analyses will be tabulated in an appendix. The standardized mean difference along with its 95% confidence interval (CI) will be used as an effect measure for the limb difference (the primary outcome). Depending on the scale of the secondary outcomes, we will use either the odds ratio (OR) or the standardized mean difference (SMD) along with its 95% CI, as we expect different studies to report similar outcomes using varying assessment tests and scales. We do not currently expect to identify time-to-event outcomes during the screening process.

A random-effects network meta-analysis will be performed for all primary and secondary outcomes if enough eligible studies are identified. A network graph will be used to visualize the connectedness for each outcome, as well as the direct and indirect comparisons. If a network meta-analysis can be performed, we will use the method based on graph theory for data synthesis [32] and the reduction of weights to account for correlation in multi-arm trials [32]. If possible, direct and indirect evidence will be compared to assess the consistency (the manifestation of transitivity) and a design-by-treatment interaction model will be used to formally assess loop consistency wherever possible [33]. We will use the between-trial heterogeneity parameter for each network meta-analysis (one parameter per network meta-analysis) to assess the agreement between the randomized and non-randomized studies in the analysis [34]. We will use the design-adjusted analysis that down-weights non-randomized evidence as a sensitivity analysis [34]. If not enough data is available for one or more endpoints, we will perform random-effects pairwise meta-analyses instead, using inverse variance weighting and restricted maximum likelihood estimation. Between-trial heterogeneity will be assessed using the *τ*^2^ statistics as well as the *I*^2^ and the (generalized) *Q*-statistic. Summary of finding (SoF) tables, Forest plots (grouped by treatment contrast for network meta-analyses), and league tables will be used to visualize estimation results. We will use *P*-scores to provide ranking of treatments for each outcome [35], which will be used to visualize estimation results. Comparison adjusted funnel plots along with Egger’s regression tests will be used to assess small-study effects for all outcomes that we identify ten or more contributing studies for [36].

All analyses will be performed in R (version 4.0.3 or higher, R Foundation for Statistical Computing, Vienna, Austria) using the extensions netmeta [37] and meta [38]. Results will be considered significant at a level of 5%.

Sensitivity analyses will be performed using the design-adjusted approach for combining randomized and non-randomized evidence [34], as well as for the risk of bias and the study design. Subgroup analyses will be performed for the expected categorical effect modifiers stage condition, population, and lymphedema supermicrosurgery, if sufficient data per subgroup is available.

The results of the systematic review and network meta-analysis will be displayed graphically and the resulting level of evidence will be determined according to the Oxford Centre for Evidence-Based Medicine [39]. Recommendations will be made based on the Grading of Recommendations Assessment, Development and Evaluation (GRADE) system and the Confidence in Network Meta-Analysis (CINeMA) approach [40, 41].

## Discussion

Lymphedema prevalence is high in both developed and developing countries. In the former, the increasing incidence of secondary lymphedema is due to more effective oncological treatments and a higher number of patients surviving cancer. The incidence of lymphedema will vary depending on the nature of the primary tumor but is frequently associated with head and neck cancer (75%), melanoma (16%), urological (11%), and gynecological (37%) malignancies [42–44]. Breast cancer surviving patients are the major iatrogenic source of lymphedema since the overall risk of developing breast cancer in the European female population was estimated to be 12.9% [45] and 10–40% of them will develop a SL in the first 3 years after surgery [1].

Despite conservative strategies are available and have proven to be effective [46], these do not address the root cause for lymphedema. In an attempt to refunctionalize an injured lymphatic system, supermicrosurgical strategies like lymphovenous anastomosis and lymph node transfer have emerged. For lymphedema supermicrosurgery, the literature continues to accumulate for competing management interventions and technical variations, without strong evidence for its efficacy. Recently, a systematic intervention review analyzed the evidence of surgical intervention for the prevention or treatment of breast-related secondary lymphedema [47]. Despite providing an excellent methodology, the study design contemplated very strict and ambitious inclusion criteria, leading to the eligibility of only two studies out of 828 initial records (0.24%) [47]. To be included, the studies must fulfill the following criteria: to be a RCT limited to breast-cancer-related lymphedema and present pre-defined objective criteria for measuring of tissue volume and distribution, limb composition, and imaging tests [47]. The main drawback of this systematic review was, in our opinion, the decoupling of the expectation of ideal interventional studies for the surgical treatment of lymphedema and the reality of the body of evidence generated so far. It is debatable if the conclusions about the interventions of one of the included RCT of 36 participants is a better orientation for clinicians than the dozen of non-randomized series of hundreds of participants published so far.

To our knowledge, only Basta et al. and Carl et al. have reviewed the overall evidence of the surgical treatment of lymphedema [48, 49]. The first study was based on observational data until 2013, using unconventional methodology, and a study design that did not incorporate any recommended strategy to asses or avoid bias. The second review, more recent, study presented extensive results and a good discussion. Nonetheless, the method section was nearly non-existent and omitted all the methodological recommendations from PRISMA and the Cochrane group. As a result of these shortcomings, plastic surgeons worldwide are still unable to provide evidence-based recommendations to their patients with lymphedema. This gap of evidence was identified and confirmed by a recent review of lymphedema guidelines [50].

The primary aim of this systematic review and network meta-analysis is to synthesize the full body of evidence regarding the supermicrosurgical treatment alternatives to permit the development of individualized surgical strategies and guidelines for lymphedema surgery. The results of this review will quantify and statistically analyze the mean or median volume reductions of supermicrosurgically intervened limbs compared to conservative therapy, no treatment, and/or other supermicrosurgical interventions. Whenever possible, outcomes like quality of life, limb function, erysipelas rates, and postoperative complications will be pooled and analyzed. If the evidence is categoric enough, recommendations concerning supermicrosurgical interventions will be made in relation to the etiology of lymphedema and the clinical stages. On the other hand, if the results do not permit any endorsements, the data will be displayed for its interpretation and the analysis will be merely descriptive.

A major strength of our planned systematic review and network meta-analysis is a robust design. Besides the systematic review of Markkula et al. [47], this is the first study in this regard that will adhere to the methodological gold standard of the recommendations from PRISMA and the Cochrane group. The data gathering of several databases and the privation of artificially introduced exclusion criteria will greatly improve the sensitivity of the research. To counter a related reduction in the search precision and inclusion of non-relevant articles into the analysis, the PICOS strategy was formulated by an interdisciplinary team with a focus in avoidance of selection bias and rigorous assessment of the bias in the included trials. Besides that, the methodology contemplates to calculate the heterogeneity and study the publication bias of the incorporated studies. Furthermore, all the reviewing authors are experienced in the field and the presented study protocol was developed and refined during several meetings and extensive discussions in a multidisciplinary commission.

A possible limitation of the intended review is the inclusion of studies lacking a control group, like case series. The inclusion of non-randomized studies was decided after a pilot search in PubMed and is founded in the intention to avoid false conclusions based exclusively on the analysis of a few possible underpowered or biased RCTs. The intervened groups and their subpopulations will be pooled mathematically risking to merge heterogeneous patient populations and surgeries subtypes. To minimize the error of heterogenous pooling, the reviewing authors therefore pre-specified condition, populations, and lymphedema supermicrosurgery subgroups in advance for the data extraction and analysis.

We are confident that the intended systematic review and network meta-analysis will be an exceptional contribution to the field of reconstructive plastic microsurgery. Whether the results will allow the generation of specific guidelines and recommendations for the surgical treatment of lymphedema, or identify the particular fields where more quality studies are required, this effort will be a critical contribution to evidence-based care of patients with lymphedema.

## Supplementary Information

**Additional file 1.** PRISMA-P 2015 checklist.

## Data Availability

No data generated so far.
